# The association between paraspinal muscle parameters and vertebral pedicle microstructure in patients undergoing lumbar fusion surgery

**DOI:** 10.1007/s00264-022-05659-9

**Published:** 2022-12-23

**Authors:** Maximilian Muellner, Erika Chiapparelli, Henryk Haffer, Yusuke Dodo, Stephan N. Salzmann, Dominik Adl Amini, Manuel Moser, Jiaqi Zhu, John A. Carrino, Ek T. Tan, Jennifer Shue, Andrew A. Sama, Frank P. Cammisa, Federico P. Girardi, Alexander P. Hughes

**Affiliations:** 1 Spine Care Institute, Hospital for Special Surgery, Weill Cornell Medicine, New York City, NY, USA; 2 Center for Musculoskeletal Surgery, Charité - Universitätsmedizin Berlin, Corporate Member of Freie Universität Berlin, Humboldt-Universität Zu Berlin, Berlin, Germany; 3 Division of Orthopaedics, Department of Orthopaedics and Trauma Surgery, Medical University of Vienna, 1090 Vienna, Austria; 4 Department of Spine Surgery, Lucerne Cantonal Hospital, Lucerne, Switzerland; 5 Department of Radiology and Imaging, Hospital for Special Surgery, New York City, NY, USA

**Keywords:** Bone quality, Bone microstructure, Sarcopenia, Osteosarcopenia, Muscle morphology, Spinal fusion, Micro computed tomography, microCT

## Abstract

**Purpose:**

Lumbar fusion surgery has become a standard procedure in spine surgery and commonly includes the posterior placement of pedicle screws. Bone quality is a crucial factor that affects pedicle screw purchase. However, the relationship between paraspinal muscles and the bone quality of the pedicle is unknown. The aim of the study was to determine the relationship between paraspinal muscles and the ex vivo bony microstructure of the lumbar pedicle.

**Methods:**

Prospectively, collected data of patients undergoing posterior lumbar fusion for degenerative spinal conditions was analyzed. Pre-operative lumbar magnetic resonance imaging (MRI) scans were evaluated for a quantitative assessment of the cross-sectional area (CSA), functional cross-sectional area (fCSA), and the proportion of intramuscular fat (FI) for the psoas muscle and the posterior paraspinal muscles (PPM) at L4. Intra-operative bone biopsies of the lumbar pedicle were obtained and analyzed with microcomputed tomography (μCT) scans. The following cortical (Cort) and trabecular (Trab) bone parameters were assessed: bone volume fraction (BV/TV), trabecular number (Tb.N), trabecular thickness (Tb. Th), connectivity density (CD), bone-specific surface (BS/BV), apparent density (AD), and tissue mineral density (TMD).

**Results:**

A total of 26 patients with a mean age of 59.1 years and a mean BMI of 29.8 kg/m^2^ were analyzed. fCSA_PPM_ showed significant positive correlations with BV/TV_Trab_ (*ρ* = 0.610; *p* < 0.001), CD_Trab_ (*ρ* = 0.679; *p* < 0.001), Tb.N_Trab_ (*ρ* = 0.522; *p* = 0.006), Tb.Th_Trab_ (*ρ* = 0.415; *p* = 0.035), and AD_Trab_ (*ρ* = 0.514; *p* = 0.007). Cortical bone parameters also demonstrated a significant positive correlation with fCSA _PPM_ (BV/TV_Cort_: *ρ* = 0.584; *p* = 0.002; AD _Cort_: *ρ* = 0.519; *p* = 0.007). FI_Psoas_ was negatively correlated with TMD_Cort_ (*ρ* = − 0.622; *p* < 0.001).

**Conclusion:**

This study highlights the close interactions between the bone microstructure of the lumbar pedicle and the paraspinal muscle morphology. These findings give us further insights into the interaction between the lumbar pedicle microstructure and paraspinal muscles.

## Introduction

The aging population has a significant impact on our health care system and poses a challenge in the treatment of orthopaedic patients [1, 2]. Muscle atrophy and osteoporosis are commonly observed during aging and are known risk factors for poor outcomes after spinal fusion surgery [2–4]. Spinal fusion surgery is a common procedure in which the instrumentation of spinal segments is used to correct various degenerative pathologies [5]. Due to reduced bone quality, which is an essential determinant of implant stability, implant failure can occur that can necessitate revision surgery [3, 6, 7]. The pedicle, in which pedicle screws are mainly anchored, contributes about 60% of the pull-out strength and 80% of the craniocaudal stiffness [8]. Anchoring takes place primarily in the trabecular and subcortical bone [9]. This highlights the importance of the vertebral pedicle in spine surgery. The extent to which aging processes affect the pedicle and the vertebral body is unclear [8]. Nevertheless, bone mineral density (BMD) and pedicle cortex thickness are decreased in osteoporotic patients compared to healthy individuals [8].

Paraspinal muscles, such as the erector spinae, the multifidus, and the psoas muscles, are crucial for maintaining an upright posture and contribute to the stability of the spinal column. The posterior paraspinal muscles (PPM) are attached to the arch and the processes of the vertebrae, while the vertebral body is mainly responsible for bearing weight. Due to its position, the pedicle transfers all forces from the paraspinal muscles to the vertebral body, reflecting its unique biomechanical role [10]. This is also reflected in the distinctly higher BMD of the pedicle compared to the vertebral body, which has the lowest BMD of the vertebral structures [11, 12]. The interaction between bone and muscle is often simplified and usually limited to examining mechanical coupling through their structural attachment [13, 14]. Besides mechanical effects, there are also biochemical processes between these two tissues. As an example, it has been shown recently that muscles have an important endocrine function, influencing bone metabolism via myokines [13, 14].

In recent years, bone microstructure has become an increasingly important focus of scientific research since it plays a critical role in the stability and strength of the bone macrostructure [15–19]. Although many studies have been conducted to determine the properties of screw anchorage in the pedicle, only a few have studied its microarchitecture [19, 20]. The relationship between paraspinal muscle morphology and the bony microstructure of the lumbar pedicle has not been investigated. Therefore, our aim was to determine the correlation between the composition of paraspinal muscles and the ex vivo microstructure of lumbar pedicles using magnetic resonance imaging (MRI) analysis and micro-computed tomography (μCT) scans.

## Materials and methods

### Subjects

A prospective study on patients undergoing lumbar fusion for degenerative spinal conditions was conducted at a single academic institution from 2014 to 2017. The investigation was approved by the institutional review board, and patients gave their written informed consent. The conducted study is in compliance with the Helsinki Declaration. Patients over 18 years of age scheduled for posterior open lumbar fusion surgery with an obtained lumbar pedicle bone biopsy were considered.

**Fig. 1:**
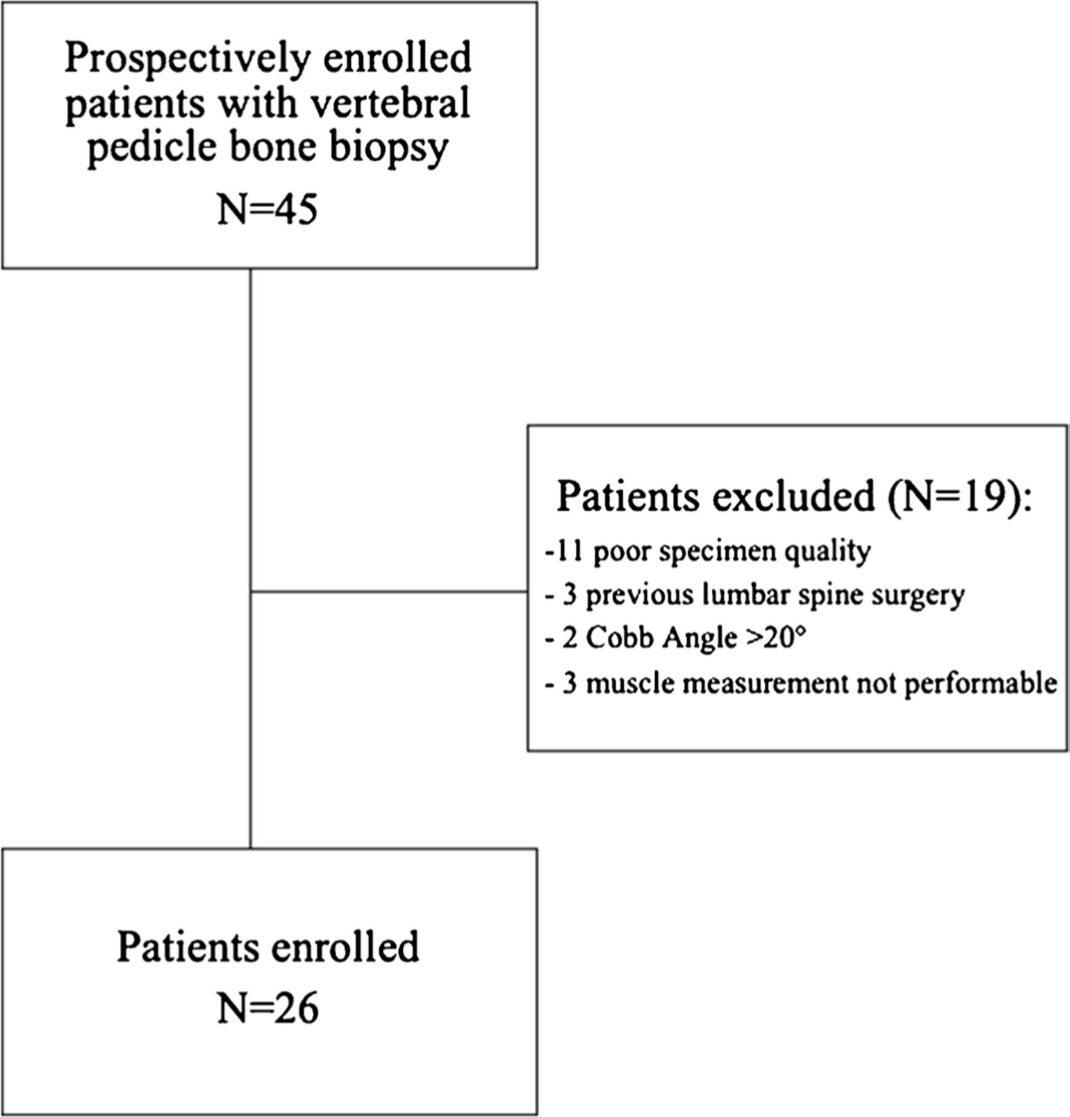
Flow chart of patient inclusion and exclusion

To reduce the impact of any previous spine surgery on bone and muscle measurements, only patients without any history of lumbar spinal surgery were included in the final analysis. Furthermore, patients with poor quality or unanalyzable bone biopsy specimens were excluded as well as patients with a lumbar Cobb Angle > 20° because of expected asymmetric degeneration of muscles (Fig. 1). A total of 45 patients were enrolled in this study; after applying the exclusion criteria, 19 had to be omitted. The reasons were poor specimen quality in 11 cases; three had previous lumbar spine surgery; two patients with a Cobb angle > 20°, and in three patients, the muscle measurements were not performable.

### Muscle measurements

**Fig. 2:**
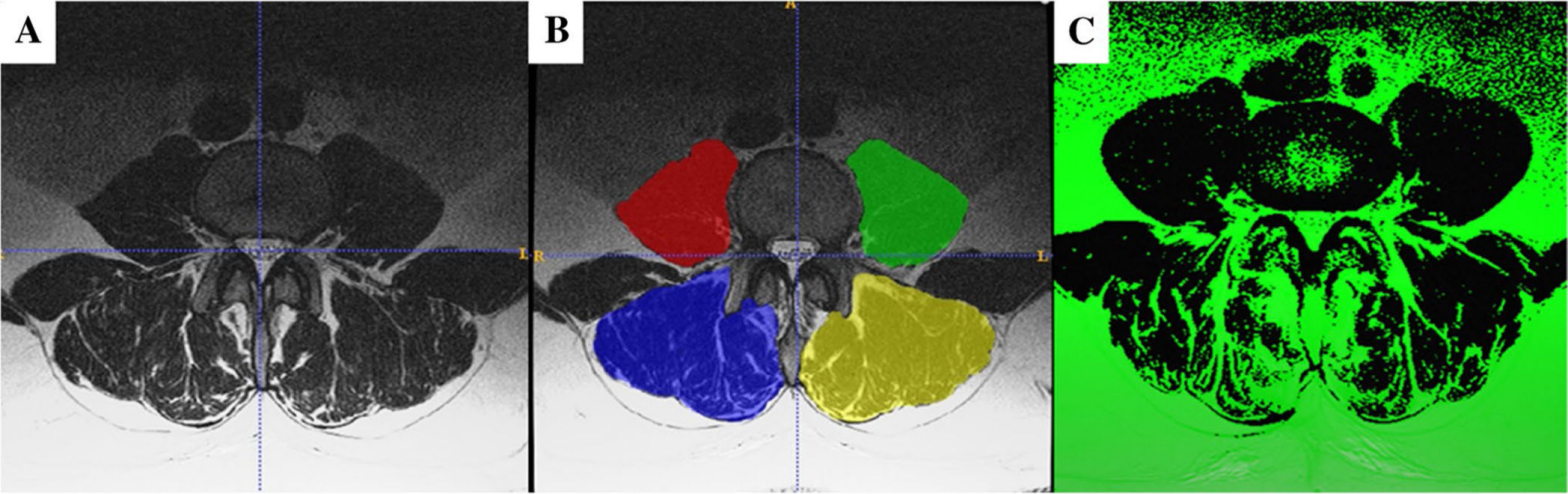
Illustration of the muscle measurement method. **A** T2-weighted axial MRI image at L4. **B** Segmentation of the area of interest where red/green is the psoas, and blue/yellow is the posterior paraspinal musculature (erector spinae and multifidus). **C** Pixels above the threshold that are interpreted as fat are shown in green

Using a dedicated software program (ITK SNAP version 3.8.0; www.itksnap.org; [21]), segmentation of both the right and left paraspinal muscles at the level of the upper endplate of L4 was performed on pre-operative axial T2-weighted MRI images. The paraspinals were segmented as follows: left and right psoas muscles and left and right posterior paraspinal muscles (PPM; combined erector spinae and multifidus muscle) (Fig. 2A and B). Previous studies have shown that ITK SNAP segmentation software has excellent intra- and interrater reliability [21].

After segmentation, another software program (Matlab^™^ version R2019a, The MathWorks, Inc., Natick, MA, USA) was used to measure the pixel intensity thresholds for the selected muscles (Fig. 2C). Pixels above the threshold were interpreted as fat, and pixels below the threshold were interpreted as muscle. These outputs included the total cross-sectional area (CSA), the functional cross-sectional area (fCSA), which was the area below the threshold, and the fat area (FAT) above the threshold. The left and right sides of the muscle groups were summarized for the total CSA, the fCSA, and the FAT and normalized by the patient’s height (cm^2^/m^2^). The percentage of fatty infiltration of muscles was calculated according to the formula (*Area over the threshold÷Total Area*)∗100. A detailed description of the calculated muscle parameters is shown in Supplemental Table 1. All measurements were performed by an orthopedic spine research fellow in his residency training. The measurement method has been described previously and demonstrated excellent intra- and interrater reliability [22].

### Acquisition of bone biopsy specimens

**Fig. 3:**
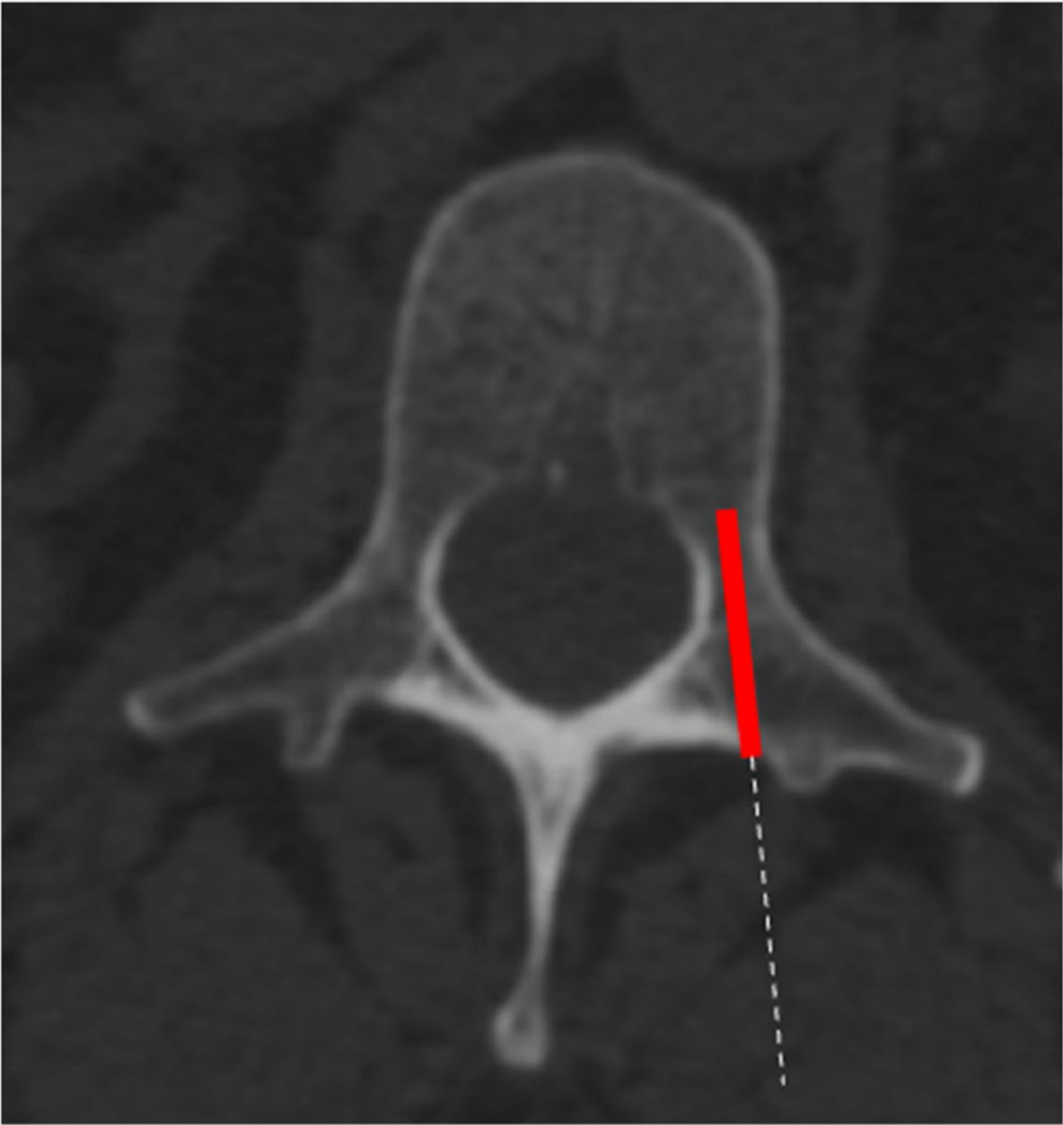
Acquisition of the bone biopsies. The red box indicates the area of the bone biopsy collection. The dashed line represents the ideal access route for the biopsy

The patients were placed in a prone and true anterior–posterior position. After the skin incision and the usual surgical steps, the anatomical conditions were checked by fluoroscopy. The bone acquisition was performed before the drilling for the screws. The bone biopsies were all performed using a similar technique using a Jamshidi bone trochar. The biopsies were taken from the left L4 or L5 pedicle (Fig. 3). The correct position of collecting the specimen was confirmed by multiplanar fluoroscopy. All the bone specimens were around 4 mm in diameter. All biopsies were processed in the same way after harvesting. The bone samples were fixed in ethanol and embedded in polymethylmethacrylate (PMMA). The specimens were taken by board-certified senior spinal surgeons with expertise in spinal fusion surgery.

### Micro‑computed tomography (μCT)

The μCT scans were performed with a Scanco μCT35 system (Scanco Medical, Bassersdorf, Switzerland) with a voxel size of 10 μm, an energy of 55 KVp, and 145 μA with an integration time of 400 ms per view. Scans were performed in a 75% ethanol solution. Scanco μCT software (HP, DEC windows Motif 1.6) was used for 3D reconstruction and image viewing. After 3D reconstruction, volumes were thresholded using a global threshold of 280 Hounsfield units (HU) was set. The threshold was set in order to separate grey values from the bone. Specimens’ regions of interest were generated by defining the respective contours on sequential reconstructed volume slices for the trabecular and cortical area. The collected trabecular parameters were total volume (TV), bone volume (BV), bone volume fraction (BV/TV), connectivity density (CD), structure model index (SMI), trabecular number (Tb.N), trabecular thickness (Tb.Th), trabecular separation (Tb.Sp), tissue mineral density (TMD), specific bone surface (BS/BV), and an apparent density (AD). The measured cortical parameters were total volume (TV), bone volume (BV), bone volume fraction (BV/TV), tissue mineral density (TMD), and an apparent density (AD).

A detailed description of the collected parameters is shown in Supplemental Table 2.

### Statistical analysis

The Shapiro–Wilk test was applied to check the data for normal distribution. Means and standard deviation (SD) or median and interquartile range [IQR] are reported depending on whether the data was normally or not normally distributed. To perform comparisons within two groups, the Mann–Whitney U test for continuous variables was applied. Comparisons between categorical variables were performed using Fisher’s exact test. Spearman’s correlation coefficients were calculated to determine the relationship between the μCT measurements and muscle parameters. Statistical significance was defined as *p*-value < 0.05. All statistical analyses were conducted using SPSS Version 28.0 (IBM Corporation, New York, USA).

## Results

A total of 26 prospectively enrolled patients (13 females, 13 males) with a mean age of 59.1 ± 12.1 years and a mean BMI of 29.5 ± 5.8 kg/m^2^ were included in the final analysis. No significant differences were seen between the sexes with regard to the patient characteristics collected. Table 1 summarizes patient characteristics.

### Muscle measurements

All muscle measurements normalized by patient height and stratified by biological sex are shown in Table 2. Significant differences were seen between the sexes for the total CSA and fCSA of the psoas muscle, but not for the PPM. Furthermore, there was no significant sex difference with respect to the FAT and FI in the psoas muscle but also in the PPM.

### Micro‑computed tomography measurements

The μCT parameters of the cortical bone revealed no significant differences when comparing males to females. Likewise, the investigated trabecular bone microstructure parameters did not reveal any significant sex-specific differences (Table 3).

### Correlations between muscle and bone measurements

Significant correlations were found for both the PPM and psoas with μCT parameters (Table 4). However, the correlation of the psoas muscle was limited to the cortical bone only, where a significant negative correlation between TMD_Cort_ and FI_Psoas_ as well as FAT_Psoas_ was found. Furthermore, FI_Psoas_ was significant negatively associated with AD_Cortical_. There was a significant positive correlation for FAT_PPM_ and FI_PPM_ with TMD_Cort_. Only the CSA_PPM_ and fCSA_PPM_ revealed significant positive correlations with both cortical and trabecular μCT parameters. A negative association is observed between FI_PPM_ and CD_Trab_.

## Discussion

To our knowledge, this is the first study to evaluate ex vivo lumbar pedicle bony microstructure and furthermore compare it with paraspinal muscle morphology. Multiple moderate to good correlations were found between the posterior paraspinal muscles and the trabecular and cortical microstructure of the lumbar pedicle. The significant positive associations between fCSA_PPM_ with BV/TV_Cort_ (*ρ* = 0.584), BV/TV_Trab_ (*ρ* = 0.610), Tb.N (*ρ* = 0.522), and Tb.Th (*ρ* = 0.415) demonstrate the close interaction between PPM and the bony pedicle microstructure.

In addition to the importance of the pedicle in spinal surgery where it serves as an anchor for the pedicle screws, all muscular forces of the paraspinal muscles are transferred to the vertebral body through the pedicle. A cadaver study has shown that the BMD of the vertebral body differs significantly from other structures such as the lamina or the pedicle [11]. Furthermore, the bone microarchitecture that contributes to bone strength differs between the pedicle and the vertebral body [16, 17].

Besides microarchitectural differences in the cortical and trabecular bone, there are macroscopic differences that can affect bone’s biomechanical properties [17]. There are various techniques used to measure BMD, which is considered a surrogate marker for bone strength [17]. However, BMD measured by dual-energy x-ray absorptiometry (DEXA) is only responsible for about 60% of the variation in bone fragility [23]. Other parameters like bone microarchitecture and bone composition cannot be predicted with the current gold standard for evaluating BMD and DEXA [17]. Pumberger et al. demonstrated that DEXA measurements of the spine do not properly represent the microstructure of the vertebral body and are therefore not a reliable tool to determine bone quality in the spine [24].

There is the hypothesis that all bony structures are subject to similar aging processes. However, it was previously demonstrated that the BMD of the vertebral body could not be used to predict the bone volume fraction of the pedicle, which is a parameter related to the apparent density of bone [18, 25]. Inceoglu et al. demonstrated that although the BMD of the vertebral body and pedicle was significantly correlated, there was no statistically significant relationship between the BMD of the vertebral body and the bone volume fraction of the pedicle (*R*^2^ = 0.15) [18]. Therefore, it can be assumed that different factors may affect the pedicle compared to the vertebral body.

A frequently stated risk factor for osteoporosis and reduced bone quality is age. Inceoglu et al. examined the pedicle isthmus of eight human cadavers and did not detect aging effects on the trabecular microarchitecture of the pedicle [18]. The mineral content of the bone is a major factor in its mechanical properties. [26] However, if a certain mineral value is exceeded, the bone becomes more brittle [27] and probably also leads to a deterioration in toughness [28]. As demonstrated by Currey et al., there is increased mineralization of the cortical bone with age [28]. The increased cortical bone mineralization leads to a mismatch of the ratio between highly mineralized and less mineralized bone that results in increased bony homogeneity [15]. The more homogeneous the bone is, the more likely cracks and small fractures can occur, leading to reduced toughness [15].

In addition to bone degeneration, the loss of musculature plays an important role in the aging population. The loss of musculature is associated with an increased tendency to sustain falls and a reduced quality of life and decreased strength [29]. Osteoporosis and sarcopenia can coexist, a condition referred to as “osteosarcopenia.” The development of osteosarcopenia is multifactorial and is based, for example, on muscle-bone crosstalks through, among other things, myokines [30]. On the other hand, other pathophysiological processes in the context of aging play a crucial role, such as the reduced sensitivity of the musculoskeletal apparatus to utilize proteins and vitamins, resulting in catabolic processes [30]. Both diseases have an additive effect such that the increased risk of falls leads to an increased incidence and possibly severity of fractures due to osteoporosis [31]. The interaction between muscles and bones is often considered biomechanical, but there are also biochemical interactions that should be considered [14]. Our results suggest that there is a relationship between the pedicle and paraspinal musculature, although we cannot state whether the associations are due to biochemical or biomechanical effects. The most common method of representing bone mineral density is by using DEXA; it is possible to convert the areal BMD values obtained into the apparent density [25]. Our data indicates that the fCSA correlates significantly with cortical and trabecular apparent density, whereas age is not associated with trabecular μCT parameters. As the fCSA is considered a surrogate for muscle strength, this may suggest that the muscle strength of the PPM may have a direct impact on the microarchitecture of the pedicle. However, the positive relationship between PPM and microstructural elements of trabecular bone in our study is in line with previous results [32, 33].

Interestingly, only the posterior paraspinal muscles showed a positive correlation with cortical and trabecular bone. However, the FI_PPM_ and FAT_PPM_ revealed a positive correlation with cortical TMD. Curiously, the FI and FAT of the PPM and psoas muscle had the opposite correlation to the cortical TMD. The assumption would be that both muscle groups should have a similar influence on TMD_Cort_ due to mechanical coupling. However, the difference may be due to the fact that the PPM and the psoas have different attachment points, resulting in a different function on the lumbar spine.

In our study, a negative correlation was shown between FI_Psoas_ and TMD_Cort_. It has been shown that visceral fat and subcutaneous fat have a contrary effect on bone density, whereas visceral fat has a negative impact on bone density [34]. Previously, it has been demonstrated that muscle attenuation of the psoas muscle is inversely correlated with the amount of visceral fat [35]. A lower muscle attenuation indicates an increased fat infiltration of the muscle [36]. It is possible that the correlation difference in fatty infiltration of the psoas muscle and PPM on TMD_Cort_ may be due to the retroperitoneal location of the psoas. Due to the spatial proximity, a possible hypothesis could be that visceral fat has a similar effect on bone as psoas fat.

The psoas muscle is relatively poorly understood to date but has recently received increased attention by research in various fields. A previous study demonstrated a different aging pattern for the psoas muscle and the quadriceps femoris muscle in women [37]. Increased postoperative morbidity and mortality as well as poorer outcomes after surgery have been associated with lower psoas muscle size normalized by height [38–40]. However, these investigations did not examine the bone quality. A recent study by Stanuszek et al. concluded that muscle quality is more important than its mere size represented by CSA in patients treated for lumbar discopathy [41]. However, the extent to which this may be related to TMD_Cort_ cannot be conclusively determined at this time, and further investigations need to be conducted. Moreover, our results support the findings of Turcotte et al. that demonstrated in a prospective study the positive influence of physical exercise on the CSA of the paraspinals and BMD of the vertebrae [42]. Other investigations have demonstrated that muscle is a protective factor in the development of adjacent segment disease after spinal fusion [43, 44]. This further highlights the importance of muscle measurements in identifying patients potentially at risk pre-operatively.

There are several limitations of this study that should be considered. First, our sample size was small, and it was a cross-sectional study. A longitudinal study design with a larger study population could address these issues and may elucidate how the two tissues interact over time, also enabling multivariable analyses with adjustments for potential confounders. However, a longitudinal design is likely to be impractical as the bone morphology data could only be collected intra-operatively. Moreover, all patients in the study underwent posterior spinal fusion surgery. Further studies in unaffected participants and covering different age groups need to be performed to validate our results. Nevertheless, we provide the first data of ex vivo pedicle bone biopsies analyzed with μCT and further analyzed the paraspinal muscle morphology.

In conclusion, these results offer new insights into the association between paraspinal musculature and the microarchitecture of the vertebral pedicle. We demonstrated the importance of the fCSA of PPMs on the bony microstructure of the lumbar pedicle. The results may indicate that through the strengthening of the PPM, there is a chance to decelerate the progression of pedicle bone loss and improve spinal surgery outcomes by reducing the potential risk of pedicle screw loosening.

## Supplementary Material

Supplementary Material

## Figures and Tables

**Table 1:** Patient demographics

	All	Female	Male	*p*-value
N	26	13	13	-
Age [years]^a^	59.1 ± 12.1	57.4 ± 10.6	60.8 ± 13.4	0.139
BMI [kg/m^2^]^b^	29.5 ± 5.8	26.6 ± 5.1	32.1 ± 5.9	0.064
Diagnosis* *N* (%) Spinal stenosis	24 (92.3)	12 (92.3)	12 (92.3)	1.000
Foraminal stenosis	0 (0)	0 (0)	0 (0)	n/a
DDD	25 (96.2)	12 (92.3)	13 (100)	1.000
Spondylolisthesis	26 (100)	13 (100)	13 (100)	n/a
Neurogenic claudication	2 (7.7)	1 (7.7)	1 (7.7)	1.000
Herniated nucleus pulposus	1 (3.8)	1 (7.7)	0 (0)	1.000
Treated segments *N* (%)				
I	13 (50)	8 (61.5)	5 (38.4)	0.543
II	10 (38.5)	4 (30.8)	6 (46.2)	
III	3 (11.5)	1 (7.7)	2 (15.4)	
Bone status L1/2 vBMD [mg/cm^3^]^b^	124.9 ± 38.7	129.9 ± 38.2	120.3 ± 40.1	0.547
Osteopenia/osteoporosis [≤ 120 mg/cm^3^] *N* (%)	13 (50)	6 (46.2)	7 (53.8)	1.000
ASA-score *N* (%)				
I	2 (7.7)	2 (15.4)	0 (0)	0.532
II	13 (50)	6 (46.2)	7 (53.8)	
III	11 (42.3)	5 (38.4)	6 (46.2)	
Comorbidities *N* (%) Diabetes mellitus	1 (3.8)	1 (7.7)	0 (0)	1.000
COPD	2 (7.7)	1 (7.7)	1 (7.7)	1.000
Hypertension	11 (42.3)	5 (38.4)	6 (46.2)	0.695
Congestive heart failure	0 (0)	0 (0)	0 (0)	n/a
Active smoker	13 (50)	6 (46.2)	7 (53.8)	1.000

a Median and interquartile range is given due to non-normal distribution

b Mean and the standard deviation is presented

* Some patients had multiple symptoms

*BMI*, body mass index; *DDD*, degenerative disc disease; *vBMD*, volumetric bone mineral density; *ASA*, American Society of Anesthesiology, *COPD*, chronic obstructive pulmonary disease

**Table 2:** Muscle measurement results

	*Muscle Parameters*				
		All *N* = 26	Female *N* = 26	Male *N* = 26	*p*-value
*Psoas*	CSA [cm^2^/m^2^]	8.5 ± 2.5	6.9 ± 1.6	10.0 ± 2.4	**< 0.001**
	fCSA [cm^2^/m^2^]	7.8 ± 2.4	6.3 ± 1.5	9.3 ± 2.3	**< 0.001**
	FAT [cm^2^/m^2^]	0.7 ± 0.6	0.6 ± 0.4	0.8 ± 0.9	0.577
	FI [%]	8.3 ± 6.6	9.1 ± 5.7	7.5 ± 7.6	0.568
*PPM*	CSA [cm^2^/m^2^]	19.4 ± 3.1	19.5 ± 3.7	19.4 ± 2.5	0.951
	fCSA [cm^2^/m^2^]	11.4 ± 2.6	11.6 ± 2.7	11.2 ± 2.5	0.692
	FAT [cm^2^/m^2^]	8.1 ± 2.1	7.9 ± 2.1	8.2 ± 2.2	0.699
	FI [%]	41.5 ± 9.8	40.6 ± 8.5	42.4 ± 11.1	0.636

Mean + standard deviation is presented. Significant values are in bold. Statistical significance was defined as a *p*-value < 0.05. *PPM*, posterior paraspinal muscles; *CSA*, cross-sectional area; fCSA, functional cross-sectional area; *FAT*, the total area of fat; *FI*, percentage of fat in total muscle

**Table 3:** Results from micro-computed tomography (μCT) for cortical and trabecular bone overall and sex-specific

	*Bone parameters*				
		All *N* = 26	Female *N* = 13	Male *N* = 13	*p*-value
Cortical Bone	BV/TV [%]	67.3 ± 20.3	62.0 ± 23.3	72.6 ± 15.9	0.187
	TMD [mg/cm^3^]	862.1 ± 37.4	853.2 ± 28.3	871.1 ± 44.0	0.230
	AD [mg/cm^3^]^a^	621.1 [477.2; 726.0]	612.8 [406.3; 706.7]	668.4 [577.1; 764.8]	0.264
Trabecular Bone	BV/TV [%]	37.5 ± 10.6	34.4 ± 8.4	40.5 ± 12.0	0.145
	CD [1/mm^3^]	98.3 ± 66.2	91.9 ± 70.8	104.7 ± 63.1	0.630
	SMI	1.4 ± 1.0	1.2 ± 0.5	1.6 ± 1.3	0.321
	Tb.N [1/mm]	3.5 ± 0.9	3.3 ± 1.0	3.6 ± 0.9	0.384
	Tb.Th [mm]	0.17 ± 0.04	0.16 ± 0.04	0.17 ± 0.04	0.467
	Tb.Sp [mm]	0.36 ± 0.09	0.37 ± 0.1	0.35 ± 0.08	0.703
	TMD [mg/cm^3^]^a^	810.2 ± 36.6	805.3 ± 27.0	815.0 ± 44.8	0.507
	AD [mg/cm^3^]^a^	30.5.9 [255.6; 356.3]	258.1 [228.8; 336.1]	320.6 [269.0; 413.7]	0.204
	BS/BV [mm^2^/mm^3^]^a^	18.5 [15.1; 22.4]	19.0 [16.1; 46.8]	17.7 [13.8; 22.8]	0.336

a Median and interquartile range is given due to non-normal distribution. All other parameters are given as mean ± standard deviation. Significant values are marked as bold. Statistical significance was defined as a *p*-value < 0.05*. BV/TV*, bone volume fraction; *CD*, connectivity density, *SMI*, structure model index, *Tb.N*, trabecular number; *Tb.Th*, trabecular thickness, *Tb.Sp*, trabecular separation; *TMD*, tissue mineral density; *AD*, apparent density; *BS/BV*, specific bone surface.

**Table 4:** Correlation between muscle and μCT parameters

*Association between muscular parameters and the bone measurements*									
		CSA *PPM*	fCSA *PPM*	FAT *PPM*	FI *PPM*	CSA *Psoas*	fCSA *Psoas*	FAT *Psoas*	FI *Psoas*
*Cortical*	BV/TV [%]	**0.590** **	**0.584** **	0.151	− 0.075	0.018	0.159	− 0.290	− 0.372
	TMD [mg/cm^3^]	**0.439** *	− 0.010	**0.602** **	**0.538** **	0.052	0.253	− 0.555**	− 0.622**
	AD [mg/cm^3^]	**0.586** **	**0.519** **	0.231	0.017	− 0.009	0.154	− 0.378	− 0.444*
*Trabecular*	BV/TV [%]	**0.607** **	**0.610** **	0.198	− 0.140	0.102	0.185	− 0.152	− 0.282
	CD [1/mm^3^]	**0.482** *	**0.679** **	− 0.124	− 0.418*	0.139	0.111	− 0.024	− 0.118
	SMI	0.127	0.155	− 0.098	− 0.092	− 0.350	− 0.282	− 0.135	0.009
	Tb.N [1/mm]	**0.437** *	**0.522** **	− 0.050	− 0.292	0.199	0.199	− 0.061	− 0.167
	Tb.Th [mm]	0.416*	**0.415** *	0.145	− 0.119	− 0.210	− 0.043	− 0.254	− 0.279
	Tb.Sp [mm]	− 0.333	− 0.417*	0.112	0.296	− 0.048	− 0.032	0.091	0.122
	TMD [mg/cm^3^]	0.057	− 0.097	0.242	0.241	0.025	0.153	− 0.139	− 0.200
	AD [mg/cm^3^]	**0.597** **	**0.514** **	0.268	0.039	0.032	0.142	− 0.164	− 0.276
	BS/BV [mm^2^/mm^3^]	− 0.221	− 0.037	− 0.412*	− 0.234	0.052	− 0.089	0.285	0.383

Significant values are in bold. *ρ*-values marked with

* are significant at the 0.05 level. *ρ*-values marked with

** are significant at the 0.01 level. *BV/TV*, bone volume fraction; *CD*, connectivity density; *SMI*, structure model index; *Tb.N*, trabecular number; *Tb.Th*, trabecular thickness; *Tb. Sp*, trabecular separation; *TMD*, tissue mineral density; *AD*, apparent density; *BS/BV*, specific bone surface; *CSA*, cross-sectional area; *fCSA*, functional cross-sectional area; *FAT*, the total area of fat; *FI*, percentage of fat in total muscle

## Data Availability

The datasets generated and analyzed during the current study are available from the corresponding author upon reasonable request.
