# Supplementary material for: The association between paraspinal muscle parameters and vertebral pedicle microstructure in patients undergoing lumbar fusion surgery
Source: Int Orthop. Author manuscript; Available in PMC 2023 Nov 21. (PMC10661537; doi:10.1007/s00264-022-05659-9)
Supplement: Supplementary Material [file NIHMS1936929-supplement-Supplementary_Material.docx]

Supplemental Material

| Abbreviation | Variable | Description | Unit | Normalized by patients height |
| --- | --- | --- | --- | --- |
| CSA | Cross sectional Area | Total muscle Area | cm^2^ | cm^2^/m^2^ |
| fCSA | Functional cross-sectional Area | Lean Muscle Area | cm^2^ | cm^2^/m^2^ |
| FAT | Fat Area | Area of Fat | cm^2^ | cm^2^/m^2^ |
| FI | Cross sectional Area divided by Fat area | Percentage fat in muscle | % | % |

**Table 1.** Description of the muscle parameters collected.

| Abbreviation | Variable | Description | Unit |
| --- | --- | --- | --- |
| BV | Bone Volume | Bone volume of the selected region | mm^3^ |
| TV | Total Volume | Total volume of the selected region | mm^3^ |
| BV/TV | Bone volume fraction | Proportion of bone to the total volume in the region of interest | % |
| CD | Connectivity density | Measure of the degree of connections of the trabecular bone normalized by total volume | 1/mm^3^ |
| AD | Apparent density | Mass of mineralized tissue in relation to total volume | mg/cm^3^ |
| Tb.N | Trabecular number | Average number of trabeculae per unit length | 1/mm |
| Tb.Th | Trabecular thickness | Average thickness of the trabecula | mm |
| Tb.Sp | Trabecular separation | Average distance between the trabeculae | mm |
| TMD | Total mineral density | Density measurement limited to the volume of the calcified bone tissue and excluding the surrounding soft tissue | mg/cm^3^ |
| SMI | Structure model index | Gives information about the structure of the trabeculae and ranges from 0-3 (0 stands for parallel plates and 3 for cylindrical rods) | Unitless |
| BS/BV | Specific bone surface | Ratio of segmented bone surface area to bone volume | mm^2^/cm^3^ |

**Table 2.** Description of the parameters collected by µCT(42)
